# Book Review

**Published:** 1900-12

**Authors:** 


**Tropical Diseases.** By Patrick Manson, C.M.G., M.D. Revised and Enlarged [Second] Edition. Pp. xx., 684. London: Cassell and Company, Limited. 1900.—The appearance of an enlarged and revised edition of this work within two years is fully justified by the rapid strides made in our knowledge of the ætiology of some of the diseases of tropical climates, and although the size of the book is increased by 58 pages it still keeps its manual form, and its value as a text-book is beyond question. The author, in his introduction, remarks that a student of medicine should also be a naturalist; and the truth of this is evident when we come to study the more recent researches on the parasitic origin of malaria and other diseases. Nevertheless, the subject of malarial parasitism is so clearly dealt with in this volume, that the reader will find no great difficulty in mastering the essentials of the mode of propagation of this disease through the agency of the mosquito, the discovery of which is due to the combined labours of Dr. Manson and Dr. R. Ross. The definition given of malaria is significant of the complete change in our knowledge of the causation of this disease. Simply and modestly the author shows the reasoning by which he was led to the conclusion that the parasite of malaria in passing from one host to another must have an extra-corporeal existence; and his researches on filarial disease induced him to advance the opinion that some species of mosquito was the intermediate host; an opinion subsequently confirmed by the able researches of Dr. R. Ross. Chapter II. is of great value to the student who desires to familiarise himself with the appearance of the parasite, and if the directions there given be followed, no great difficulty should be experienced in demonstrating the various forms of its intra-corporeal existence; in fact the author, in his remarks on the diagnosis of malaria, considers that in some cases the only *certain* test is a microscopical examination of the blood. In the treatment and prophylaxis of malaria, no drug, in the author's opinion, replaces quinine; and his teaching as to its administration, either by mouth or hypodermically, is thoroughly practical. The probable germ origin of yellow fever is shortly discussed. As regards its treatment, Dr. Manson puts careful nursing in the first place. The chapters in Section III., dealing with cholera, dysentery, tropical liver and liver abscess, are amongst the most valuable in the book. The author inclines to the belief that both dysentery and liver abscess are due to the amæba coli. The detail of the variety of symptoms of liver abscess is most excellent, and the possibility of large collections of pus being altogether overlooked is impressed on the reader. Leprosy, Yaws, Oriental sore, Sprue, Animal parasites, and other strange and rare affections met with in the tropics, are all treated in a practical and scientific manner. We have no hesitation in saying that this book should be in the possession of all medical men whose practice is in tropical countries.

**Rheumatism, Rheumatoid Arthritis, and Subcutaneous Nodules.** By C. O. Hawthorne, M.D. Pp. ix., 53. London: J. & A. Churchill. 1900.—This essay is an endeavour to face the question whether the deformed condition of the joints in rheumatoid arthritis is due to the same agency as that which produces the acute polyarthritis of rheumatic fever. Cases are adduced which qualify to some extent the statement that subcutaneous nodules are conclusive evidence of rheumatism, and give some evidence against the view that the occurrence of such tumours in undoubted cases of rheumatoid arthritis is a demonstration of the rheumatic nature of that affection. The apparent contradictions which the facts disclose forbid any dogmatic declaration in a field which is much embarrassed by personal creeds.

**Human Nature: its Principles and the Principles of Physiognomy.** By Physicist. Part II. Pp. viii., 175. London: J. & A. Churchill. 1899.—For those who wish to matriculate for Colney Hatch this book may be strongly recommended. As we have no wish to enter that excellent institution, we did not study the book from beginning to end, but contented ourselves with the state of mental confusion produced by reading the first hundred pages or so. In fact we were rather alarmed (being of short stature and limited means) to find (on page 17) the following statement: "Of two healthy individuals, the purpose . . . of the larger will incline . . . towards Self Preservation, and the purpose of the smaller will incline . . . towards Reproduction." Also it is disconcerting to find (on page 59) that chronic dyspepsia is characterised by "great piety and morality, avarice, love of admiration (Vanity), and the desire of knowledge." This is almost sufficient to induce one to cultivate chronic dyspepsia. But confusing as the book is, it is difficult to get much amusement out of it, and it cannot be recommended for the ordinary reader.

**The Pathogenesis and Treatment of Cancer without Operation.** By Robert Bell, M.D. Pp. 43. Glasgow: Robert Love Holmes. 1900.—Much as we should rejoice to think that the author has really found a means of curing cancer by the application of hot air and thyroid extract, we are not convinced by the meagre report of cases given at the end of a book which appears to be written more for the lay than the medical mind. That thyroid extract is helpful we may admit, but the present state of our knowledge and the common sense of the profession will, we trust, prevent others from imitating the temerity of one who has "no hesitation in going the length of affirming, that where complete removal of the disease can reasonably be expected by surgical measures, the therapeutics, which I advocate, will prove quite as efficacious. . . ." It would be well for Dr. Bell to have the specimens of his cancers obtained by curettage submitted to some independent pathologist.

**A Manual of Surgery.** By William Rose, M.B., and Albert Carless. Third Edition. Pp. xiv., 1182. London: Baillière, Tindall, and Cox. 1900.—This excellent work has so rapidly run through its three editions that there is little change in the text of this edition, though some new plates and skiagrams have been added. There has, however, been a welcome change in the arrangement of the matter. Formerly injuries and diseases of the different regions were often widely separated; but now they have been brought together, and reference is thereby much facilitated. As new editions appear there is frequently a tendency to increase the size of a book, but we are pleased to notice that since its first issue the volume has only been increased by twenty pages. The whole work is praiseworthy and reliable, clearly written, well abreast of the times, and worthy of recommendation.

**A Contribution to the Surgery of Fractures and Dislocations of the Upper Extremity.** By J. E. Platt. Pp. x., 228. London: H. K. Lewis. 1899.—This is a record of all the fractures and dislocations of the upper extremity seen at the Manchester Infirmary during a period of two years. The book is full of long tabulations relating to the nature of the fracture, displacement, treatment, and result, etc., etc., which are very tedious to look at. There are a few skiagrams showing various fractures, but these for the most part are not very good. The book represents much careful work; but of course a large number of very ordinary fractures and very ordinary modes of treatment are included in it, which can be studied equally well in the modern text-books of surgery. In fact, except for the author's own edification, we doubt if much value will accrue from the work.

**Enlargement of the Prostate: its Treatment and Radical Cure.** By C. Mansell Moullin, M.D. Second Edition. Pp. xi., 211. London: H. K. Lewis. 1899.—In the present issue space has been gained by wisely omitting some of the obsolete operations described in the former edition. In suitable cases the author prefers orchidectomy to division of the vasa deferentia, and gives good reason for his choice; but the views of Mr. Reginald Harrison on this subject have not received the consideration they deserve. The book is written in clear language, and forms one of the best treatises on a disease which is common enough, but which is only too apt to be neglected from ignorance of what can be done in such cases. We heartily commend the work to the general practitioner as well as to the operating surgeon.

**A Synopsis of the British Pharmacopœia, 1898.** By H. Wippell Gadd. Fifth Edition. Pp. 223. Bristol: Evans, Gadd & Co. 1900.—The fact that this is the fifth edition of a work, of which over 6000 copies have been sold within two years of its publication, is a sufficient indication of its utility.

**A Manual of Pathology.** By JOSEPH COATS, M.D. Fourth Edition, revised throughout by LEWIS R. SUTHERLAND, M.B. Pp. xxv., 1132. London: Longmans, Green, and Co. 1900. —A melancholy interest attaches to the issue of the fourth edition of this well-known work, in that it appears after the lamented death of the learned and accomplished author. Prof. Gairdner has written a short and appreciative prefatory notice of Prof. Coats, in which he states that "the present edition may be said to have the counsel and support of the late author up to the very close of his life," and the work of revisal has been carried out on the lines laid down by him. Many of the old illustrations have been replaced by new ones, and they are, both old and new, well-chosen and abundant. The book is so well known, and has attained to such wide appreciation, that it is scarcely necessary to say more than that Dr. Sutherland in editing this edition has been careful to keep all its original features. It is difficult nowadays for a work which deals with all branches of pathology to be equally good in all, and in this one for instance the section on the malarial organism is somewhat inadequate; but taken as a whole the book still gives the most complete account of pathology available for the student in one volume of moderate size, and never fails to keep before him its bearings upon practical medicine.

**Lowestoft as a Winter Resort.** Pp. 31. Lowestoft: Dotesio & Todd. 1899.—The information contained in the thirty-one pages of this booklet is accurate and interesting. It is too often forgotten that the East Coast, justly celebrated for its March winds, is equally conspicuous for its mild and sunny autumn; and the fisherman's saying that "you can warm your hands in the sea up to Christmas" gives the clue to the reason of the daily range of temperature being less at Lowestoft than at Greenwich. Golf, shooting, fishing, and yachting provide plenty of outdoor attractions, and the town appears to be quite up to date in its indoor amusements.

**Golden Rules of Ophthalmic Practice.** By GUSTAVUS HARTRIDGE. Pp. 69. Bristol: John Wright & Co. [1900].—Though intended primarily for the student, this little book contains much information which should be of value to the general practitioner. The subjects are arranged in alphabetical order, and the information given is wonderfully complete considering the limited amount of space at the disposal of the author. We can very strongly recommend the book to the notice of students working in ophthalmic departments.

**Mentally-Deficient Children: Their Treatment and Training.** By G. E. SHUTTLEWORTH, M.D. Second Edition. Pp. xvi., 180. London: H. K. Lewis. 1900.—The appearance of a second edition of this work sufficiently indicates that it has found acceptance with the profession, as indeed it deserved to do, for it gives a clear account of a class of cases on which every practitioner is sometimes appealed to for advice. The sections on treatment, training, and education of mentally-defective children are particularly helpful. To this second edition two additional chapters have been added; one on the results of an investigation by the Education Department, and another on the practical measures adopted for special instruction of the feeble-minded by several school authorities.

**On the Prevention of Eye Accidents Occurring in Trades.** By SIMEON SNELL. Pp. 32. London: John Bale, Sons & Danielsson, Ltd. 1899.—The writer, who is eminently qualified to express an opinion on the subject, gives figures and statistics to prove the awful frequency with which eye accidents occur, more especially in the iron and steel trades. He states that it is not the employers but the men who are reluctant to adopt precautionary measures! The conclusions arrived at are that grinders should wear glass protectors, and that the use of protectors should be made compulsory to iron and steel workers. For these latter he recommends protecting spectacles or shields made of aluminium gauze, which will not rust and cause very little interference with sight.

**Golden Rules of Obstetric Practice.** By W. E. FOTHERGILL, M.D. Pp. 71. Bristol: John Wright & Co. [1898].—While we do not as a rule approve of the use of such works as the above, we must admit that Dr. Fothergill has embodied in his little book a large number of useful hints, many of which are not found in the larger text-books. A work of this nature is necessarily brief to baldness, and should not be looked upon as more than a waistcoat-pocket remembrancer. As far as it goes the information contained in it is accurate and well put, and if used properly may be found to be of considerable value. It must not, however, be looked upon as a substitute for the larger manuals, but used merely as an adjunct to them.

**Lectures on Hæmorrhage and Eclampsia.** By ROBERT JARDINE, M.D. Pp. 76. Edinburgh: William F. Clay. 1899.—The author gives a very interesting description of numerous cases treated by him. One special point to be noted is the use he makes of intra-cellular injections of saline fluid in both conditions—in the cases of hemorrhage to re-establish the circulation, and in the cases of eclampsia to promote diuresis. In spite of the fact that this treatment was not always successful, we feel no doubt as to the author's contention in favour of the method, especially in the second class of cases, since we have found similar results from the use of continuous rectal irrigation with saline solution. The mortality of both conditions is so high that the fact of the line of treatment proving a failure should not influence us unfavourably, since the rapidity of the loss of blood in severe hemorrhage is such that it is difficult to be sufficiently prompt in its application, while in eclampsia the damage to the kidney is often enough to prevent any re-establishment of its excretory power. It is interesting to note that one case of eclampsia treated with morphia died with, apparently, symptoms of morphia poisoning. In a fatal case of accidental hemorrhage, in which Champetier de Ribes's bag was used, the bag burst, and before this evidently detached the placenta, thus aggravating the trouble. We have always felt that this accident (detachment of the placenta) was a possibility in cases treated by this instrument, and prefer methods not so liable to cause this accident, such as the use of conical plugs of iodoform gauze or the dilating bags of Barnes. The lectures are thoroughly practical and lucid: we can strongly recommend their perusal to the practitioner. We notice with pleasure the beneficial result of blood-letting in some of the cases of eclampsia; this method of treatment has fallen into unmerited disuse, and in properly selected cases its effects are marvellous. We notice among some few clerical errors the use of decidu-æ for decidua. Some capital plates are introduced to illustrate various cases. The lectures are a thoroughly practical and useful contribution to the study of the complications referred to.

**The Diagnosis and Treatment of Eczema.** By Tom Robinson, M.D. Second Edition. Pp. 136. London: J. & A. Churchill. 1900.—The term eczematous diathesis which is used by Mr. Robinson (if there is such a condition) might, we think, have been expressed with more brevity: as, for instance, Mr. Jonathan Hutchinson's way of putting it, namely, that some people possess skins which do not wear well. The cases Mr. Robinson says he has been seeing during the last few weeks, with "an affection of the face which is usually known as *relapsing erysipelas*, which displays an erythematous condition of the bridge of the nose, spreading with more or less symmetry over the malar bones and going to the ears," etc., strike us as unusually resembling cases of lupus erythematosus. We dislike the word erysipelas being used unless it has been proved to originate with the specific agent of the disease, namely, the *streptococcus pyogenes*, with which the *streptococcus erysipelatis* appears to be identical. Mr. Robinson says emphatically that eczema is not contagious. Unna's view is as emphatic in the opposite direction. We think it should be more clearly stated that tinea cruris seu axillaris always depends upon the ringworm fungus, and constitutes the so-called eczema marginatum. To aver that the cases which are known as pityriasis rubra are examples of erythematous eczema is, to say the least, a bold assertion for a dermatologist. Mr. Robinson says that when eczema appears during the process of teething, it is absurd to think that it is produced or modified by it. We are not so sure of this. We believe Mr. Robinson is not far from correct in asserting that herpes zoster only occurs once during a lifetime. Mr. Robinson thinks the itching accompanying lichen ruber is less than occurs in eczema. In the treatment of chronic eczema, Mr. Robinson is a believer in the efficacy of arsenic, if the eczema occurs on both sides of the body. We think it asoften does harm as good, especially if it disturbs the disgestive organs. The local treatment, as well as the general management, is practical and common-sense treatment. Mr. Robinson says we must never send eczematous patients to the sea. We consider there are very many exceptions to this rule. He well says experience must be the great teacher, whether it be in recognising disease or in guiding it to recovery. The book is in large type and on good paper, and will no doubt be found useful to practitioners and students of medicine; but it cannot be compared to the modern text-books of dermatology.

**Spencer's Disease: Dermatitis Multiformis Exfoliativa.** By WALTER SPENCER, M.D. Pp. 16. London: Pewtress & Co. 1899.—We have looked through this pamphlet with interest and care, but we fail to see why such a distinct appellation should be applied. The name of a practitioner labelled upon any disease or manifestation of unusual symptoms cannot be too strongly deprecated. Dr. Spencer relates twenty-five cases of infectious dermatitis, and occurring, with one exception—case 1, E. K., aged 3½ years—amongst newly-born children. In two cases, Nos. 7 and 20, a rash was present at birth. Some of the cases had admittedly a syphilitic taint. We feel sure we have had similar experiences of a desquamating complaint, which is certainly not scarlet fever nor measles nor German measles, although our cases were probably in older children than in Dr. Spencer's. We are strongly of opinion that there is an infectious disease, which is distinct from the above-mentioned exanthems, and when recovered from gives no immunity whatever from them. The low temperature, not exceeding 101°—except in one fatal case, No. 23, where it reached 103°,—is quite in accordance with our experience, and we have met with quite extensive desquamation continuing for six or seven weeks; and we also believe that morbilli and rubella may be regarded as predisposing agents: they give the germs, which are sui generis, a better breeding ground. We are quite prepared to hear of more and more cases as time passes on, and practitioners are on the alert for differentiation.

**Surgical Ward Work and Nursing.** By ALEXANDER MILES, M.D. Second Edition. Pp. viii., 288. London: The Scientific Press, Limited. 1899.—We are not enchanted with this work; in fact, it is little else than an illustrated instrument catalogue interlarded with some letterpress, and the latter does not contain the practical information one expects in a book of the sort. For instance, the description of Thomas' hip-splint is most inadequate for one who has never seen or heard of it before, and it is for such as these the book is intended. The statement on page 64, that "it is quite exceptional to find a hospital hypodermic syringe fit for use," makes us wonder where the author studied, as we have always found a number at our hospital ready and those in the anæsthetic room never to fail. Much of the material in this book is, in our opinion, learnt properly by the student only practically and not from any book at all; and if a book is necessary, there are better ones than this.

**Medical Missions.** By G. E. F. M. Pp. 40. Weston-super-Mare: Davies Brothers.—One chief object of this pamphlet is to point out the very great extent to which the New Testament history is a record of medical mission work, and that we have the same injunction to heal diseases as to evangelise nations. Attention is also drawn to the fact that a medical missionary is a fully-qualified specially-trained medical man, and to the extent to which medical missions assist in the spread of civilisation.

**Transactions of the American Dermatological Association for 1898 and 1899.** Concord, N.H.: The Rumford Press. 1899-1900.—The presidential address of the 1898 meeting was given by Dr. J. Nevins Hyde, of Chicago, and contained many interesting statements. Lavage of the colon and aseptic treatment of the alimentary canal for acne were highly spoken of. An International Board of Health to deal with a problem like leprosy was suggested; but at present we fear there is little likelihood of this bearing practical fruit, although an International Leprosy Conference has been held and 'broken ground.' Segregation for lupus and other disfiguring face diseases may become a problem in the future. Congenital origin seems to be receiving a broader foundation. Tubercular bacilli have been found in the testes of tuberculous patients without other uro-genital symptoms. Artificial tuberculosis of the placenta of guinea-pigs has resulted in the birth of tuberculous offspring. It is interesting, but at the same time rather humiliating, to have it recorded that 278 new remedies during the year (past) have been placed on the chemists' lists. There ought to be no diseases left. The "contents" of the Transactions are fifteen in number, and some of them, unquestionably, of great value, in addition to reports on "Exhibition of Cases" and those of the statistical committee. "Trophic Dermatoses following Fractures," by Dr. J. Zeisler, of Chicago, opens up new ground. The volume, containing the papers read at the 1899 meeting, consists of 176 pages, as compared with 166 in 1898. The work is well printed, and the plates cleverly executed. The presidential address by Dr. J. A. Fordyce, of New York, is far-reaching and pregnant with suggestions: *e.g.* (p. 3), "the products of the bacillus of tuberculosis in the skin . . . , may differ from those produced by the organism in the lungs, bone, or lymph-nodes, and this difference may explain the variations in the clinical course of lupus vulgaris as it affects the skin and mucous membranes, and the true tuberculous ulcer of the mucous membranes and adjacent skin. The same organism previously inoculated in the skin producing its peculiar toxin gives rise to a definite clinical picture, while the inoculation resulting from the bacilli grown in the lungs, with its different or more virulent toxin, results in a condition which is quite unlike the slowly-progressing lupus." Dr. L. D. Bulkley, of New York, contributes a very valuable paper on "Imperfect or deficient urinary secretion in connection with certain diseases of the skin," the outcome of 2000 analyses of the urine of skin patients, made in his office, during the last ten years. "Two Epidemics of Alopecia Areata in an Asylum for Girls," by the Vice-President of the Association, Dr. J. T. Bowen, of New York, give strong colour to the parasitic theory of certain forms, in spite of many opinions to the contrary. Dr. Sherwell, of Brooklyn, confirms by very cogent reasons his treatment of scabies by the dry sublimed sulphur process. We can corroborate its simplicity, utility, and cleanliness. The exhibition of clinical cases, photographs, and drawings was large and good, apparently; but nomenclature is not simplified by such terms as "fibroma—dermatolysis extraordinarius Japaniensis!"

**Transactions of the Medical Society of the State of New York.** Published by the Society. 1900.—A series of seven papers on the State-care of tuberculous patients is worthy of especial consideration just now when provision for consumptives is being sought for in this direction. The papers are headed as follows:—(1) "The Duty of State and Municipality in the care of Pulmonary Tuberculosis among the Poor," by Edward O. Otis, M.D. (2) "Remarks upon the work accomplished at the State Hospital for Consumptives at Rutland, Mass.," by Vincent Y. Bowditch, M.D. (3) "Some results of the Climatic and Sanatorium Treatment of Tuberculosis in the Adirondacks," by E. R. Baldwin, M.D. (4) "The Infectious Character of Tuberculosis and the Prognosis of Incipient Consumption," by George Blumer, M.D. (5) "The Policy of the State relative to the Spread of Tuberculosis," by Enoch V. Stoddard, M.D. (6) "Legislation concerning Tuberculosis—Past, Present, and Future," by Senator Horace White. (7) "Taxation with relation to the State-care of Consumptives," by Hon. Otto Kelsey. (8) "Bovine Tuberculosis," by James Law, F.R.C.V.S. It is claimed that on the grounds of economy, prophylaxis, and humanity treatment and care should be afforded to the poor consumptive. The magnitude of the task seems to be only a greater reason why the State should undertake it. Private philanthropy has made a good beginning, but can only do a fraction of it. Whilst England has seventeen hospitals, homes, and sanatoria for consumptives, free or partially so, Germany takes the lead with more than double this number.

**The American Year-Book of Medicine and Surgery.** London: Rebman, Limited. Philadelphia: W. B. Saunders. 1900.—Gould's year-book is now issued in two volumes: that on medicine contains 656 pages, written by well-known contributors of Philadelphia, Chicago, Cleveland, Montreal, Boston, and Baltimore. An article on cerebro-spinal fever deserves especial mention at the present time, as it appears that we are now passing through an epidemic period of this disease in Europe and America. Osler, in his Cavendish lecture of last year, gives particulars of recent epidemics in Boston, Baltimore, and elsewhere, and considers the disease to be an acute infection produced by a peculiar micrococcus, the meningococcus, to which Weichselbaum has given the name diplococcus intracellularis (*vide* page 313): various authors consider this to be the specific organism, and admit no other in the etiology of the disease. Our June number (page 128) contained a report on two sporadic cases occurring in the Bristol General Hospital, and more recently several cases have been observed in the Bristol Royal Infirmary. It appears that we have something like an epidemic amongst us, and it is one of the most fatal of all acute diseases, having a mortality of about 68 per cent. One of the most accurate means of diagnosis is that described as Quincke's lumbar puncture—a simple, quite harmless procedure, which enables us to obtain a sample of cerebro-spinal fluid in which the diplococcus may be easily demonstrated. Another symptom usually present is the spasm and contraction of the flexor muscles of the knee, known as Kernig's sign. Osler also comments on the occurrence of pneumococcic meningitis, and directs attention to the resemblances between pneumonic and cerebro-spinal fever in the matter of epidemicity. A new pathognomonic sign of measles (Koplik's spots) was described by Dr. Watson Williams in our June number (page 139). A coloured plate is reproduced in the year-book (page 274), and shews these spots better than any description can do. The volume on surgery contains 560 pages, of which 220 are devoted to general surgery, and the rest to the special departments of obstetrics, gynæcology, ophthalmology, otology, rhinology, laryngology, and anatomy. The abstracts from medical and surgical papers contained in these volumes are unquestionably of great value, both to those who, in a good medical library, have access to the original papers, and to those who have not. For the former the abstracts will be used more especially as an index of the scope of articles to which they meditate referring, though in many cases the abstracts themselves will be found sufficiently full to meet their requirements.

**General Index to the Journal of the American Medical Association.** Vols. I. to XXIV. inclusive. By WILLIAM B. ATKINSON, M.D. Pp. 360. Chicago: American Medical Association Press. 1896.—This forms a companion volume to the *Index of the Transactions* of the Association; the two works thus provide a "key to the American Medical Association proceedings and papers from 1849 to 1894, and since 1883 to all medical matters that have appeared in the Journal." It has been carefully prepared and its wide usefulness is apparent. Dr. Atkinson will have not only the thanks of the present generation, but also of a grateful posterity.

**The Medical Annual Synoptical Index to Remedies and Diseases.** For the twelve years 1887 to 1898. Pp. 411. Bristol: John Wright & Co. [1899].—This is not an index merely to the subjects in the twelve volumes of the *Medical Annual* which have already been published; for under the heading of "Remedies" it contains the dose and uses of drugs, and under the heading of "Diseases" it gives the methods of treatment. There is also given a summary of the chief alterations in the new Pharmacopœia, and some test types for examining the acuteness of vision, both being reprinted from former numbers of the *Annual.* The index therefore appeals to a wider field than those who are fortunate enough to possess the back numbers of the *Medical Annual,* and is usefully arranged for ready reference on the matters of which it treats.

**The American Medical Quarterly.** Vol. I., No. 1. June, 1899. New York: The American Medical Quarterly Company.—The editor of this magazine tells us that its special province is "to furnish its readers practical rather than experimental or theoretical contributions from the front ranks of the profession." We are glad to have this new quarterly on our exchange-list. It ought to find a place in an already voluminous literature; for if the first number may be taken as a sample of the high standard of the articles which is to be maintained in future issues, the success of the *American Medical Quarterly* will be ensured. It is freely and well illustrated.

**Consumption Chart and Case Paper.** Bristol: J. Wright & Co.—The increased attention to the details of tubercular phthisis, and the necessity for recording many details not mentioned on the ordinary temperature charts, has led to the publication, at the suggestion of Dr. L. Kidd, of a special chart with arrangements for recording the temperature four times daily, and with spaces for recording weight, hæmoptysis, chest measurements, and the condition of sputum and other excreta. The case paper contains outline forms for noting the physical signs, and all the principal points are mentioned; there is, however, one singular fault, as it allows quite insufficient space for any details of the previous history of the patient.

**Edinburgh Hospital Reports.** Vol. V. Edinburgh: Young J. Pentland. 1898. Vol. VI. Edinburgh: Oliver and Boyd. 1900.—These two volumes well maintain the high reputation of their predecessors, and we may say at once are equally well printed and fully illustrated. It is impossible to adequately indicate in a short notice the contents of volumes which contain such a large number of papers covering so wide a range of subjects. Without attempting to do this we may briefly state that, besides papers of much practical interest on well-known diseases, there are some which give valuable information on many extremely rare conditions, which add to our knowledge of them, and others giving the details of original methods and researches in medicine, surgery, and pathology. We think no one can read the reports without finding something to learn and something of interest to him.

**Electro-Medical Instruments and their Management, and Illustrated Price List of Electro-Medical Apparatus.** By K. Schall. Sixth Edition. Pp. vii., 154. London: Bemrose & Sons Ltd. 1899.—The busy man who has not time to consult large works on electricity will find this book very useful to keep by him for reference; for in addition to being a very complete catalogue of electro-medical instruments, it has a treatise written in a most lucid style explaining the elements of electricity and the various electrical instruments in medical use, including a description of the Wehnelt Interrupter for X-ray work.

**Burdett's Hospitals and Charities.** London: The Scientific Press (Limited). 1900.—In welcoming this last issue of a compilation of the utmost utility to all who take a practical interest in hospitals and their management, we would direct attention to the authoritative articles on such topics as "The Financial Problem of the London Hospitals," "Hospital Construction in 1898 and 1899," "The Cost of Hospital Management, and of In- and Out-patients" Maintenance," "Home and Foreign Missions," etc., which precede the statistical sections.

**Transactions of the Association of American Physicians.** Vol. XIV. Philadelphia: Printed for the Association. 1899. —This volume contains thirty papers, varying in length and in importance, but remarkable for their general excellence and the high level sustained by the work of the Association. Many of the cases reported are distinguished by their rarity, but the chief feature of the book is the practical value of the work done in advancing the study of clinical medicine. These Transactions will always repay careful study.

**International Directory of Laryngologists and Otologists.** Compiled by Richard Lake. London: The Rebman Publishing Company (Ltd.). 1899.—A careful examination of the Directory convinces one that enormous trouble has been taken to ensure accuracy. A few errors and omissions are to be found, and this is unavoidable in a first issue; but the compiler deserves the thanks of all members of his speciality for his eminently useful production.

**Reports from the Laboratory of the Royal College of Physicians, Edinburgh.** Vol. VII. Edinburgh: Oliver and Boyd. 1900.—Forty papers appear in this volume, all records of work of a highly scientific character and many of direct value to the medical practitioner. The expense of resetting papers already in print has been avoided by merely binding together these reprints. Such papers as that on "The Intracranial Circulation in some of its Aspects," by the late George Elder, whose untimely death will be deplored by all who have been acquainted with his work; "The Action of Arsenic on the Bone-marrow and Blood," by Ralph Stockman and S. D. W. Greig; "An Experimental Investigation of the Action of Red Bone-marrow on the Blood in Anæmia," by J. S. Fowler; and others too numerous to mention, furnish evidence of the valuable work done in the laboratory.

**Journal of Surgical Technology.** July, 1900. New York: Technique Publishing Co.—This is an entirely new publication "devoted to the pure technique of surgical procedures," while it avoids questions of pathology, etiology, prognosis and the non-mechanical treatment of disease. There are some excellent original articles, but looking at the book as a whole it seems to have a somewhat limited scope and to overlap the work of the many excellent journals already in publication. The first number would, however, appear to justify its existence, and we wish the new journal every success.

**Annual and Analytical Cyclopædia of Practical Medicine.** Vol. V.—*Methyl-blue to Rabies.* Philadelphia: The F. A. Davis Company. 1900.—This volume includes many specialities—otology, laryngology, ophthalmology, neurology, pædiatrics, obstetrics, and therapeutics. Articles of especial note are Pleurisy, Catarrhal Pneumonia, and Lobar Pneumonia. The editor laments the death of two members of his associate staff—Dr. Norman Kerr, of London, and Dr. J. E. Graham, of Toronto. The article on Morphinism is the last contribution of the former to medical literature. "The special field to which Dr. Kerr devoted his labors has lost its most brilliant exponent; the profession at large one of its most faithful and honest workers." It is impossible to criticise these volumes as they succeed each other: they are on similar lines, and any individual article one may consult rarely fails to give one the desired information and assistance.

